# Current views on etiology, diagnosis, epidemiology and gene therapy of maturity onset diabetes in the young

**DOI:** 10.3389/fendo.2024.1497298

**Published:** 2025-01-20

**Authors:** Lilya U. Dzhemileva, Elena N. Zakharova, Anna O. Goncharenko, Maria V. Vorontsova, S. A. Rumyantsev, Natalia G. Mokrysheva, Marina Y. Loguinova, Vladimir P. Chekhonin

**Affiliations:** Endocrinology Research Centre, Moscow, Russia

**Keywords:** maturity-onset diabetes of the young (MODY), autosomal dominant inheritance, diabetes monogenic forms, gene therapy, AAV-vectors, European and Asian populations

## Abstract

MODY, or maturity-onset diabetes of the young, is a group of monogenic diseases characterized by autosomal dominant inheritance of a non-insulin-dependent form of diabetes that classically manifests in adolescence or in young adults under 25 years of age. MODY is a rare cause of diabetes, accounting for 1% of all cases, and is often misdiagnosed as type 1 or type 2 diabetes. It is of great importance to accurately diagnose MODY, as this allows for the most appropriate treatment of patients and facilitates early diagnosis for them and their families. This disease has a high degree of phenotypic and genetic polymorphism. The most prevalent forms of the disease are attributed to mutations in three genes: GCK (MODY 2) and (HNF)1A/4A (MODY 3 and MODY 1). The remaining MODY subtypes, which are less prevalent, have been identified by next generation sequencing (NGS) in the last decade. Mutations in the GCK gene result in asymptomatic, stable fasting hyperglycemia, which does not require specific treatment. Mutations in the HNF1A and HNF4A genes result in pancreatic β-cell dysfunction, which in turn causes hyperglycemia. This often leads to diabetic angiopathy. The most commonly prescribed drugs for the treatment of hyperglycemia are sulfonylurea derivatives. Nevertheless, with advancing age, some patients may require insulin therapy due to the development of resistance to sulfonylurea drugs. The strategy of gene therapy for monogenic forms of MODY is still an experimental approach, and it is unlikely to be widely used in the clinic due to the peculiarities of MODY structure and the high genetic polymorphism and clinical variability even within the same form of the disease. Furthermore, there is a lack of clear gene-phenotypic correlations, and there is quite satisfactory curability in the majority of patients. This review presents the main clinical and genetic characteristics and mutation spectrum of common and rarer forms of MODY, with a detailed analysis of the field of application of AVV vectors in the correction of hyperglycemia and insulin resistance.

## Introduction

Maturity-onset diabetes of the young (MODY), a historical term for some forms of monogenic diabetes, is a group of inherited non-autoimmune (antibody-negative) diabetes mellitus disorders that manifest at a young age. The prevalence is estimated to be 1/10,000 in adults and 1/23,000 in children, although the prevalence of MODY in different ethnic and racial groups may be underestimated (1–3) as studies to date have been conducted primarily in European populations (4).

**Figure 1:**
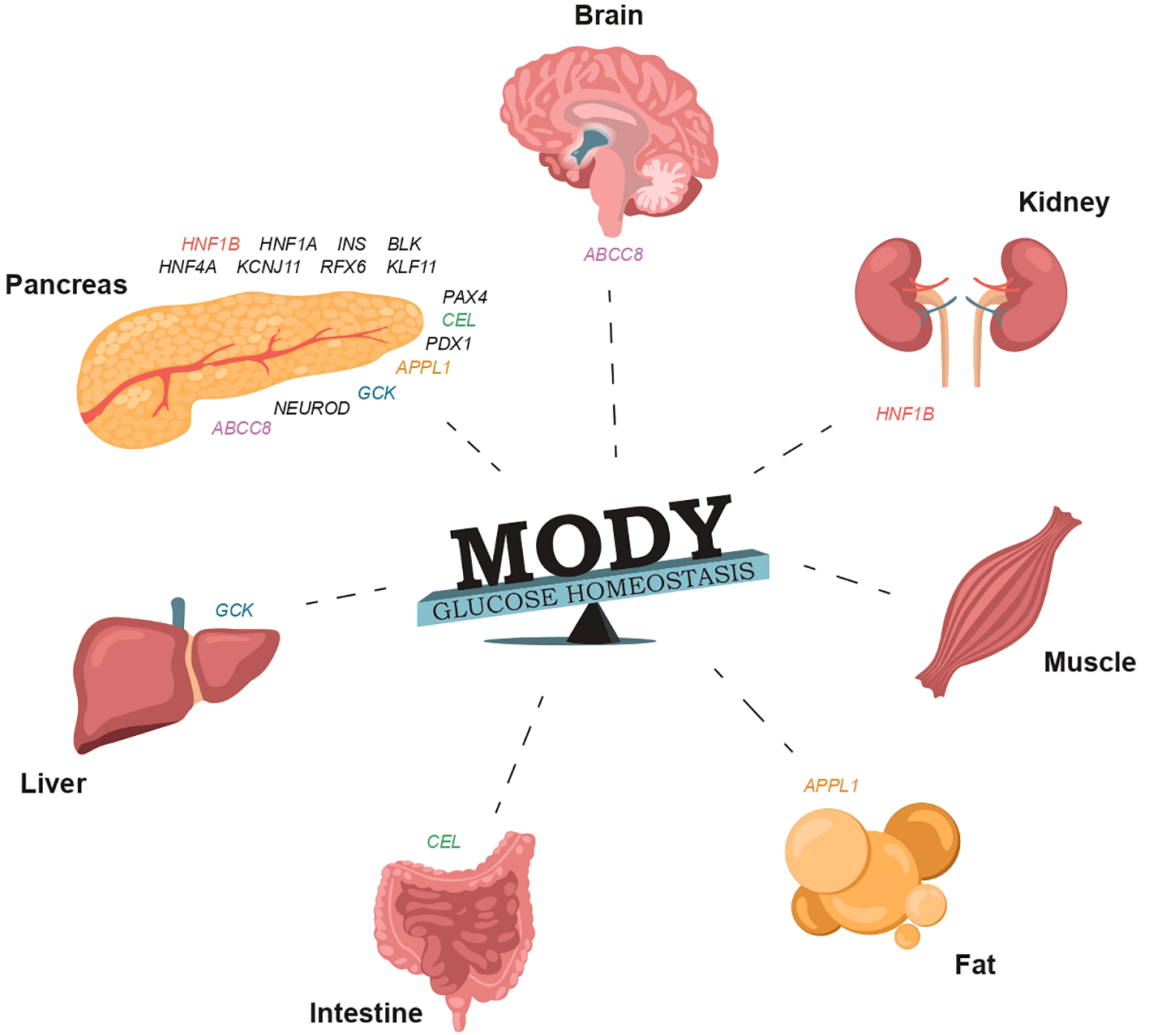
The involvement of specific genes in glucose homeostasis and the normal functioning of organs that regulate carbohydrate and lipid metabolism can result in damage that may manifest in various forms of MODY (**Supplementary Table S1**).

This “maturity-onset diabetes of the young” (MODY) is in stark contrast to the main form of insulin-dependent diabetes in young adults, which is predominantly sporadic (5). The diagnosis of MODY is typically made at a young age (i.e., under 25 years of age) when there is a paucity of autoantibodies to pancreatic islet cells, a low insulin requirement, a family history of autosomal dominant diabetes, and the absence of a history of obesity or diabetic ketoacidosis (DKA) (6). It has since been established that some patients diagnosed with MODY have a history of DKA, a positive result for pancreatic antibodies, and no family history indicating a history of diabetes in relatives (7). The aforementioned factors contribute to the emergence of specific challenges in the process of diagnosis. In the absence of a known molecular cause, MODY is often classified as a subtype of type 2 diabetes. However, since most cases of MODY currently have an inherited etiology involving defects in genes expressed in the beta cells of the pancreatic islet apparatus, MODY is best classified as a form of “another specific type of diabetes - genetic defects in beta cell function” (**Figure 1**) (8). In most populations, the prevalence of MODY is approximately 5% of patients with type 2 diabetes (9). If we analyze in detail families from European and Asian populations with MODY diagnosis, family history and absence of mutations at known loci of different forms of this diabetes, their number ranges from 15 to 20% in European families with clinical MODY and up to 80% of familial cases in Japanese people (10). Additionally, MODY is disproportionately prevalent among the Black American population. In a study of families with atypical forms of diabetes manifesting in childhood, molecular genetic testing identified MODY in nearly 75% of families. Autoantibodies to islet cells were not identified in any of the patients or their relatives examined. Additionally, HLA-DR3 and HLA-DR4 major histocompatibility complex (MHC) allele frequencies, which are known to be associated with diabetes, were not detected in probands or siblings. Furthermore, diabetes did not cosegregate with HLA haplotypes in informative families (11).

GCK-MODY(MODY2) and HNF1A-MODY(MODY3) are the two most common forms of the disease, accounting for more than 80% of patients with MODY from European populations (12, 13), whereas 80% of Japanese and Chinese patients with MODY do not have lesions in known MODY genes. In the literature, such forms of diabetes mellitus without an identified genetic cause are referred to as MODYX. It is likely that most Chinese and Japanese patients with MODY have defects in as yet unknown genes, and these forms of the disease appear to be characterized by higher insulin resistance (14, 15).

The GCK-MODY(MODY2) and HNF1A-MODY(MODY3) forms, each accounting for approximately 30% to 60% of all MODY forms, have a similar prevalence of approximately 1:1000 individuals (16); however, among all causes of MODY, the prevalence of GCK-MODY is slightly higher in some populations in the United States, Germany, Italy, France, and Spain (17).

To date, more than 20 genetic variants of monogenic MODY phenotypes have been identified, and a treatment strategy with insulin or its combination with glucose-lowering drugs from the sulfonylurea group has been defined for most forms (**Supplementary Table S1**).

Monogenic forms of diabetes are usually named after a gene, such as *HNF1A* (or HNF1A-MODY) diabetes. Some of these names are updated as new variants of the disease are discovered. The phenotypic diversity of monogenic forms of the disease highlights the high genetic polymorphism and clinical variability of MODY. An important issue is the significant genetic heterogeneity within each form of the disease. This means that there are many nucleotide sequence variants identified by GWAS and registered in the Human Gene Mutation Database (HGMD) as pathogenic, but present in the Exome Aggregation Consortium (ExAC) Genome Aggregation Database (gnomAD) at population frequencies in healthy human populations, raising many questions about their significance in disease pathogenesis as well as their persistence in the population under selection pressure during evolution. Despite their classification as orphan diseases, monogenic forms of diabetes (MODY) exhibit a high degree of similarity with type I and type II diabetes in terms of clinical presentation, diagnosis, gene therapy prospects, and diagnostic procedures. This review article explores the implications of these similarities and their relevance to clinical practice.

## MODY forms

**Figure 2:**
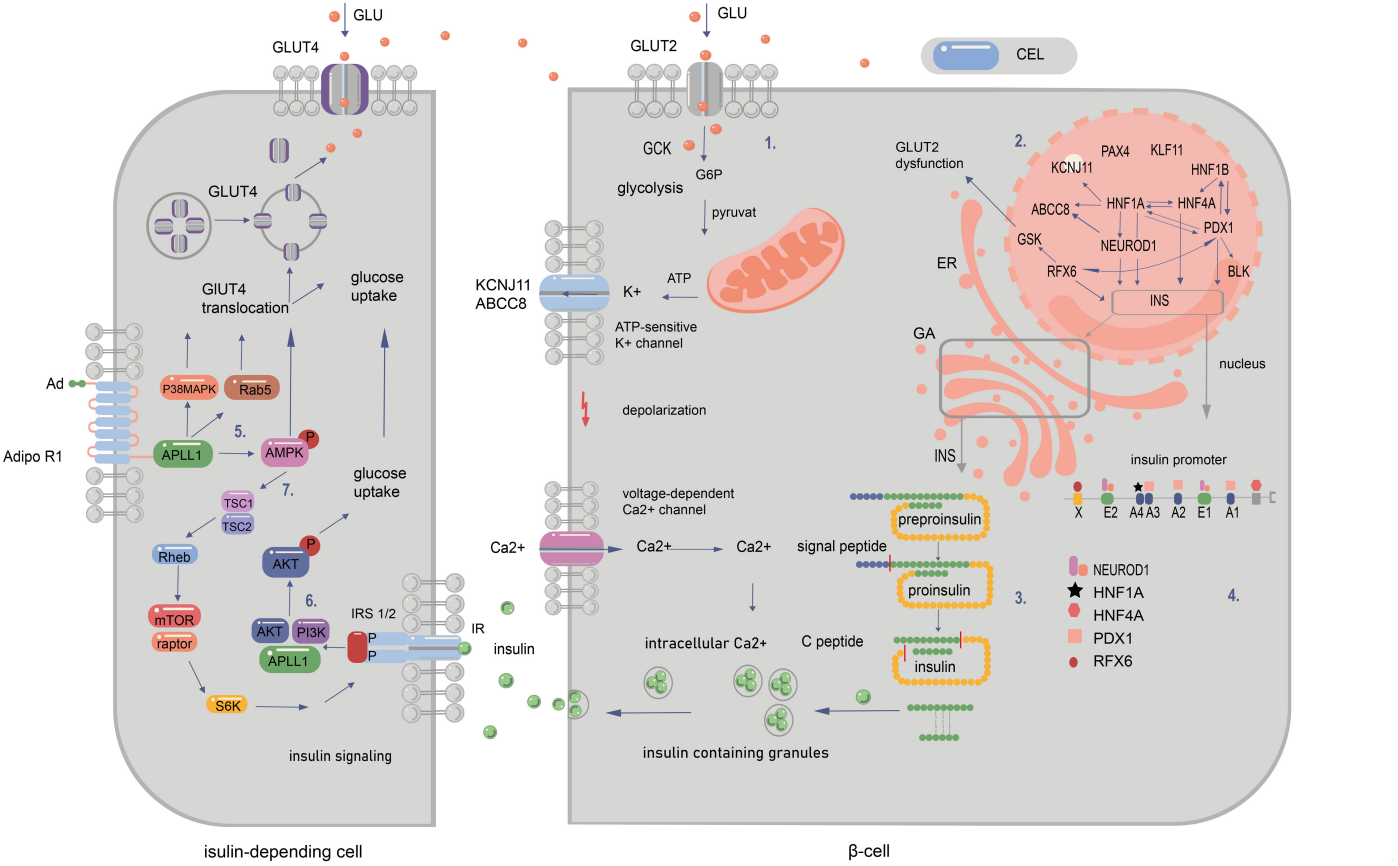
Signalling pathways in pancreatic and peripancreatic cells whose abnormalities cause diabetes of the young at maturity (MODY). 1. The influx of glucose to the β-cell through GLUT-2 facilitates the transition of glucose to glucose-6-phosphate by GCK enzyme. This results in increased ATP production in mitochondria after the entry of G6P to the Krebs cycle. The subsequent closure of ATP-sensitive potassium channels and opening of voltage-gated calcium channels permit the release of insulin from the β cell. 2. The regulation of gene expression within the nucleus is depicted by the arrows. These indicate that a specific gene is regulated by the corresponding transcription factor (TF) in mature beta cells. Additionally, the arrows illustrate that a given protein has a binding site for the corresponding TF and/or is regulated by it at some point during pancreatic development. 3. The synthesis of insulin occurs within the cytosol, where preproinsulin is produced. Subsequently, the signal peptide is cleaved by endopeptidases within the endoplasmic reticulum (ER) during the processing of preproinsulin. The final stages of proinsulin processing occur in the Golgi apparatus, including the formation of the final vesicle, the release of C-peptide from insulin within the Golgi apparatus and within the vesicles, and exocytosis. 4. The illustration depicts the binding sites of the insulin promoter to MODY-related transcription factors. The mutations in question are those that occur in the promoter region of the INS gene, which prevent transcription factors from binding to cis-elements. Another category of mutation is that which occurs in the start codon, which abolishes translation. Finally, changes in the non-translated regions of mRNA have been observed, which increase the sensitivity of the molecule to RNA degradation. MODY-related transcription factors (PDX1, NEUROD1, HNF1a, HNF4a, HNF1b, and RFX6) play a pivotal role in pancreatic development, B-cell identification, and mature B-cell function. 5. Adiponectin (Ad) binding to the extracellular COOH terminus of adiponectin receptor 1 (AdipoR1) recruits APPL1 to the intracellular NH2 terminus of AdipoR1 and activation of AMPK, p38 MAPK, and Rab5. Binding of adiponectin to AdipoR1 stimulates glucose transporter 4 (GLUT4) translocation and glucose uptake through phosphorylated AMPK, phosphorylated p38 MAPK, and Rab5. 6. Serine phosphorylation of insulin receptor substrate (IRS) proteins has been demonstrated to enhance tyrosine phosphorylation and insulin signalling, thereby activating Akt and facilitating glucose uptake. 7.The binding of adiponectin to its receptor results in the activation of AMPK, which, in turn, activates tuberous sclerosis complex 2 (TSC2). Upon activation, TSC2, in conjunction with TSC1, inhibits the activity of Ras homology enriched in brain (Rheb), thereby suppressing the activity of mammalian target of rapamycin (mTOR) and S6 kinase (S6K). The inhibition of S6K by adiponectin enhances the ability of insulin to stimulate IRS-1 tyrosine phosphorylation, which in turn leads to Akt phosphorylation and subsequent activation of the insulin signalling pathway (20, 94–99).

It can be observed that the pathophysiologic mechanisms underlying both MODY-HNF4A and MODY-HNF1A, caused by mutations in the HNF1A and HNF4A transcription factor genes, are remarkably similar. This is due to the fact that HNF4A regulates expression of HNF1A (18). Patients with *HNF4A* or *HNF1A* mutations are thought to have significantly higher plasma glucose concentrations 2 hours after glucose administration than individuals with damage to the glucokinase (MODY-2) gene (19). Hyperglycemia in patients with MODY1 and MODY3 tends to increase over time, resulting in the need for treatment with oral hypoglycemic agents or insulin. Approximately 30-40% of patients require insulin (20). Both MODY1 and MODY3 are associated with a progressive decline in insulin secretion. The most frequent cause of MODY in patients from European populations is damage to the HNF1A gene. Patients with MODY1 or MODY3 can have the full spectrum of diabetes complications: microvascular complications, often in the form of retinal angiopathy or nephropathy. Moreover, the incidence of these complications in these patients is comparable to that of similar complications in patients with type 1 or type 2 diabetes mellitus, and is also dependent on glycemic control (21). Patients with the MODY1 form of diabetes have a loss of glucose priming effect, expressed as mild hyperglycemia on insulin secretion. Both population individuals and patients with diabetes manifestations carrying mutations in the *HNF4A* gene also secrete reduced amounts of insulin in response to glucose and arginine challenge, and have impaired glucagon secretion in response to arginine challenge (22). In addition, a defect in hypoglycemia-induced pancreatic polypeptide secretion was found in individuals with mutations in the *HNF4A* gene. These data suggest that *HNF4A* protein deficiency resulting from mutations in this gene may affect the function of beta, alpha, and polypeptide cells in the pancreatic islet apparatus (23, 24). Patients with *HNF1A* gene dysfunction often have decreased renal glucose absorption and glucosuria. *HNF4A* deficiency affects triglyceride and apolipoprotein biosynthesis and is associated with a 50% decrease in serum triglyceride concentrations and a 25% decrease in serum apolipoprotein concentrations (25). Most patients with MODY1 and MODY3 diabetes have a reduced and delayed secretory response to glucose. Such a secretory response to glucose is also found in many patients with advanced insulin-dependent diabetes mellitus (IDDM) in the absence of islet cell antibodies (10, 26). In rarer other forms of MODY patients have a “hyperinsulinemic” response to glucose, which is seen in most patients with insulin-independent forms of diabetes early in the natural history of the disease (10, 27, 28). Patients with MODY1 have characteristic changes in the insulin secretory response to glucose, i.e., these patients are characterized by beta cell dysfunction, which differs from the clinical picture of MODY patients with glucokinase gene damage (MODY2) (**Figure 2**). Recently, approximately 1 in 5 rare protein-coding nucleotide variants of the *HNF1A* gene in the general population were found to cause a molecular gain of function (GOF) of this protein. These variants increase *HNF1A* transcriptional activity by up to 50% and provide a protective effect against type 2 diabetes. A meta-analysis of 12 studies revealed a significant association between *HNF1A* GOF and a reduced likelihood of developing type 2 diabetes (odds ratio [OR] = 0.77, p = 0.007). Increased *HNF1A* expression in the liver contributes to a proatherogenic serum profile, which is in part mediated by enhanced transcription of risky genotypes in the *ANGPTL3* and *PCSK9* genes. Consequently, approximately one in 300 people are carriers of a nucleotide sequence variant of the *HNF1A* gene, which protects the carriers from developing diabetes mellitus. However, it enhances hepatic secretion of atherogenic lipoproteins, thereby increasing the risk of developing non-alcoholic fatty liver disease (NAFLD) (29).

Glucokinase-related MODY2 is a common form of the disease, particularly in children with mild hyperglycemia and in women with gestational diabetes and a family history of diabetes. It has been described in people of all races and ethnicities (21, 30). To date, more than 130 MODY-2-associated mutations have been found in the glucokinase gene according to the OMIM database (31). Heterozygous glucokinase mutations are associated with a mild form of non-progressive hyperglycemia that is usually asymptomatic at the time of diagnosis and is treated with diet alone. Fasting hyperglycemia with blood glucose concentrations of 110 to 145 mg/dL and markedly impaired glucose tolerance in most affected carriers is easily detected almost immediately after birth. About 50% of female carriers have gestational diabetes during pregnancy. In addition, almost 50% of glucokinase gene mutation carriers are obese in old age. About 2% of patients with MODY2 require insulin therapy. Diabetes-related complications are rare in this form of MODY. MODY-2 is quite common in France - mutations in the GCK gene account for 56% of MODY diabetes families (32). The remaining forms are quite rare. This is especially true when analyzing familial cases of type II DM (**Supplementary Table S1**). MODY forms are arranged in the table according to their frequency of occurrence in European populations.

While a considerable amount of research has been conducted on the most prevalent MODY subtypes (MODY-1, 2, 3, and 5), the lesser-studied MODY subtypes (MODY4, 6-14) remain relatively under-researched (31, 33). The advent of next-generation sequencing (NGS) has led to the emergence of several reports of rare MODY subtypes being identified across the globe. Notably, INS-(MODY10) and ABCC8-(MODY12) mutations have been documented at relatively higher frequencies compared to other rare subtypes (34). The clinical characteristics of the rare MODY subtypes exhibited considerable clinical and genetic heterogeneity between families of MODY patients. The rarer MODY subtypes have been associated with obesity and diabetic ketoacidosis, which present a diagnostic challenge due to the rarity of these symptoms in MODY and the absence of previous descriptions of such symptoms in the clinical characteristics of frequent MODY forms. The occurrence of microvascular and macrovascular complications has been documented to a lesser extent in patients with different MODY subtypes (35). A recent hypothesis suggests that not all 14 MODY forms are true MODYs. Furthermore, the existence of some of these rarer subtypes has been called into question (**Supplementary Table S1**).

## Difficulties in diagnosing MODY

The prevalence of MODY is estimated to be between 1 and 6% of all cases of diabetes. The aforementioned data are based on the most extensively studied European populations and patient groups, which were collected according to defined selection criteria in order to identify patients for genetic testing (36–39). However, this value is likely to be an underestimate, as it is known from the literature that approximately 50 to 80% of MODY cases are misdiagnosed as type 1 (DM1) or type 2 (DM2) diabetes (1–3). Furthermore, it is not uncommon for individuals to be diagnosed with diabetes for several years before a definitive genetic diagnosis of MODY is made. Furthermore, there is a paucity of clear guidelines for screening the various forms of MODY. The decision to perform genetic testing is primarily based on the endocrinologist’s clinical judgement and is not always mandatory. Conversely, the considerable clinical heterogeneity of different MODY forms and the similarity of type I and type II diabetes mellitus features in the clinical picture of MODY render the development of a diagnostic algorithm that would be both universal enough to detect the majority of MODY cases and specific enough to avoid the unnecessarily frequent genetic testing that may lead to an erroneous increase in the diagnosis of MODY due to the detection of polymorphic variants with questionable pathogenicity in the genes of this disease a challenging endeavour (38, 40, 41). The early neonatal period, insulin independence, and autosomal dominant inheritance are traditional features of MODY (42). The age of onset is the primary distinguishing feature of MODY, which allows it to be differentiated from other forms of diabetes. Nevertheless, MODY subtypes exhibiting disparate manifestation patterns, variable penetrance, or the presence of atypical symptoms in certain instances may not fully align with the conventional diagnostic criteria (43, 44). Furthermore, in a study involving 922 families referred for MODY testing, spontaneous *de novo* mutations affecting the *GCK*, *HNF1A*, or *HNF4A* genes were reported in 11 of 150 individuals who did not have autosomal dominant inheritance of diabetes or a multigenerational family history of hyperglycemia (45). A clarification of the classical diagnostic triad may significantly enhance clinical suspicion of MODY and facilitate the selection and referral of patients with suspected genetic testing (46). The diagnostic criteria for MODY have been refined to include persistent hyperglycemia in early adulthood (usually before age 30 years), clinical features inconsistent with type 1 or type 2 diabetes mellitus (DM1, DM2), diabetes in at least one first-degree relative, evidence of residual pancreatic function, and the absence of autoimmunity to beta cells. At present, there is no concise or standardized diagnostic algorithm for MODY (43). In order to address this issue, a systematic approach to diagnosing common MODY subtypes is presented in **Supplementary Table S1**. In general, the diagnosis of MODY requires a high degree of vigilance, clinical evaluation, diabetes-specific tests, and comprehensive genetic testing (4).

Clinical evaluation and comprehensive genetic testing are employed to distinguish MODY from other types of diabetes mellitus, determine specific treatment, identify MODY mutations in family members with hyperglycemia, and reduce the risk of complications in asymptomatic family members (47). In the event that genetic testing fails to identify one of the common MODY subtypes, comprehensive genomic testing (chromosomal microarray analysis or exome sequencing) can be employed to diagnose rare pathogenic subtypes (48), characterize whole gene deletion breakpoints (49), and identify MODY in patients with clinical features suggestive of adjacent gene deletion syndrome (50, 51). The primary challenges associated with MODY can be categorized into two main areas. Firstly, the absence of specific biochemical markers in clinical practice hinders the identification of patients with MODY and the confirmation of a diagnosis of MODY with high accuracy, rather than type 2 diabetes. Secondly, the relatively high cost of genetic tests and the limited access of patients and their families to specialized genetic laboratories in most regions, remote from specialized diabetes centers, further complicates the situation.

Genetic testing is indicated in patients with suspected MODY in the presence of a diagnosis of type 1 diabetes mellitus with negative islet antibodies, with preserved beta-cell function and low insulin requirement after a partial remission phase with a positive family history or asymptomatic cases of hyperglycemia detected during routine physical examinations. In patients with type 2 diabetes mellitus, the presence of MODY is often indicated by the absence of significant obesity and the absence of black acanthosis, as well as the presence of a family history. It is recommended that testing for islet cell antibodies be conducted in all patients meeting the aforementioned criteria. Furthermore, the presence of typical syndromal characteristics of a particular type of MODY (i.e., renal cyst/dysplasia for HNF1B-MODY; stable, non-progressive, mild hyperglycemia characteristic of GCK-MODY) should also prompt specific genetic testing. The literature indicates that even the presence of obesity or black acanthosis, or the absence of a family history of diabetes, should not preclude the diagnosis of MODY.

In addition to MODY, neonatal diabetes mellitus (NDM) is a monogenic form of diabetes. This is a relatively uncommon condition, with a genetic and phenotypic heterogeneity that manifests during the first six months of a child’s life. Individuals with NDM and a debut in the first six months of life are exceedingly unlikely to exhibit specific autoantibodies to islet cells. Conversely, they are frequently found to possess a protective HLA-genotype for type I diabetes, which may suggest a higher prevalence of NDM in this age group. As indicated in the literature, NDM can exist independently or be part of a number of syndromes, including Down syndrome, Klinefelter syndrome, Turner syndrome, Wolfram syndrome, Lawrence-Moon-Bardet-Biedl syndrome, Prader-Willi syndrome, Friedreich’s ataxia, Huntington’s chorea, and porphyria (52).

The most prevalent etiology of NSD in neonates is an imprinting disorder of the *PLAGL1* gene (6q24). This gene encodes a C2H2 zinc finger protein that functions as a suppressor of cell growth. This gene is frequently methylated and silenced in cancer cells. Furthermore, overexpression of this gene during fetal development is believed to be a contributing factor in transient neonatal diabetes mellitus (TNDM). The P1 promoter downstream of this gene is imprinted, and there is preferential expression of the paternal allele in many tissues. Insulin therapy is initiated at the time of diagnosis and is continued in the event of a relapse. Macroglossia is observed in 23% of newborns (53).

Walcott-Rallison syndrome is a rare autosomal recessive disorder characterized by the persistence of insulin-dependent diabetes in the neonatal or early infancy. After the initial presentation, epiphyseal dysplasia, osteoporosis, and growth retardation may develop. Hepatic and renal dysfunction, mental disorders, and cardiovascular abnormalities are also common multisystem manifestations. It results from damage to the *EIF2AK3* gene (2p11.2). A total of 17 cases of this syndrome have been described in the literature. The syndrome is characterized by the combination of DM with epiphyseal bone dysplasia (90%), osteopenia (50%), acute liver failure (75%), developmental delay (80%), and hypothyroidism (25%). Exocrine pancreas symptoms may also occur. The age of onset is typically within the first six months of life (54, 55).

## MODY in pregnancy

The screening for gestational diabetes, although there are some differences between countries, is a standardized procedure for the detection of pregnancy-related diabetes mellitus. Nevertheless, approximately 5% of women with gestational diabetes may be affected by undiagnosed MODY, and, moreover, MODY in this context often manifests subclinically (56). It is regrettable that, at this time, there are no international guidelines that are specifically dedicated to the identification of maturity-onset diabetes of the young (MODY) among gestational forms of diabetes. Nevertheless, the differential diagnosis of MODY from gestational diabetes is of great importance, as maternal glycemic control, which necessitates specific therapeutic approaches, as well as the presence or absence of mutations in the fetus, can significantly impact the outcome of pregnancy. There is an elevated risk of macrosomia in GCK-MODY, should the fetus lack the GCK mutation from the mother, and in HNF4A-MODY, should the fetus possess the HNF4A gene mutation from the parents. Additionally, there is a potential for hyperinsulinemic hypoglycemia, particularly in offspring of parents with HNF4A-MODY. This necessitates distinct therapeutic and pharmacological strategies throughout the gestational and neonatal periods (57).

In pregnant women with a known GCK-MODY mutation, treatment recommendations are based on fetal genotype and growth. In cases where the mother has a mutation in the GCK gene and the fetus does not have a mutation in the GCK gene (or it is not implied), achieving adequate glycemic control is challenging due to the mother experiencing higher fasting and postprandial glycemic fluctuations in the first trimester compared to pregnant patients with HNF1A-MODY, despite the use of insulin therapy. This is due to the fact that administered insulin suppresses endogenous insulin secretion by the pancreas, thereby eliciting counter-regulation and resulting in a reduction in blood glucose levels. These patients require higher doses of insulin and have higher insulin needs than what would be predicted based on their replacement doses (58).

The diagnosis of HNF1A/HNF4A-MODY is considerably less prevalent among individuals with presumed gestational diabetes. These subtypes of diabetes are characterized by a progressive course, with diagnosis typically occurring well before the onset of pregnancy. HNF1A/HNF4A-MODY diabetes should be considered in a young, pregestational, obese woman with symptomatic or asymptomatic hyperglycemia without ketoacidosis and a positive family history of diabetes or a history of gestational diabetes as a potential indicator of autosomal dominant inheritance (7, 59). It is typical for there to be an absence of islet-specific antibodies and for there to be preserved endogenous insulin secretion, with detectable positive C-peptide. In cases where HNF1A-MODY is diagnosed during pregnancy, glucosuria may occur at an early stage, accompanied by relatively low glycemia. The presence of the HNF4A-MODY form should be suspected, particularly in cases with a history of abnormal gestational weight gain, macrosomia (approximately 56% of all cases), and transient hyperinsulinemic neonatal hypoglycemia (~15% of cases) in the pregnant woman herself in her neonatal period or in her offspring from a previous pregnancy. Two treatment options are currently available, although clinical data are scarce. The first option is to switch to insulin before pregnancy, which is probably the best option. The second option is to continue a sulfonylurea derivative (mainly glibenclamide, as it has the most robust evidence) in pre-conception and early pregnancy, with switching to insulin at the end of the first trimester. However, this option can only be chosen in those with HNF1A-MODY or HNF4A forms. Furthermore, this applies to patients with MODY who have achieved optimal glycemic control with sulfonylurea derivatives. It is also noteworthy that sulfonylurea preparations, along with other pharmacological classes of hypoglycemic agents, are regarded as safe during pregnancy and can be employed (59).

## The epidemiology of MODY

The estimated incidence of MODY is typically diagnosed between the second and fifth decades of life. However, the prevalence of this condition remains poorly understood due to the paucity of population-based studies in certain ethnic groups (60). To date, there is a wealth of data from various regions on the incidence of MODY. However, there is a paucity of information regarding the prevalence of different genetic variants, including rare variants, in both patients and healthy individuals from pan-ethnic populations. The minimal incidence of monogenic diabetes is estimated to be 0.2 cases per 100,000 children and young adults under 18 years of age per year (61), while the estimated incidence of MODY is approximately 2.4% in children and adolescents under 15 years of age with newly diagnosed diabetes mellitus (62). The paucity of specialist awareness regarding the prevalence of this form of diabetes in diverse global populations is attributable to several factors. Primarily, the frequent misdiagnosis of MODY due to the presence of subtle or non-specific symptoms contributes to this deficiency. Additionally, the clinical features of MODY and type 1 diabetes (DM1) and type 2 diabetes (DM2) are often analogous, further complicating the distinction between the two conditions. The third salient factor is the absence of clear criteria for comprehensive genetic testing, which is further compounded by a lack of physician awareness regarding MODY.

Estimates of the prevalence of MODY subtypes in patients from northern, western, and central Europe are roughly similar: frequency data from Germany (63, 64), the Netherlands (65), Poland (65), Norway (66) and the United Kingdom are comparable (65).

**Figure 3:**
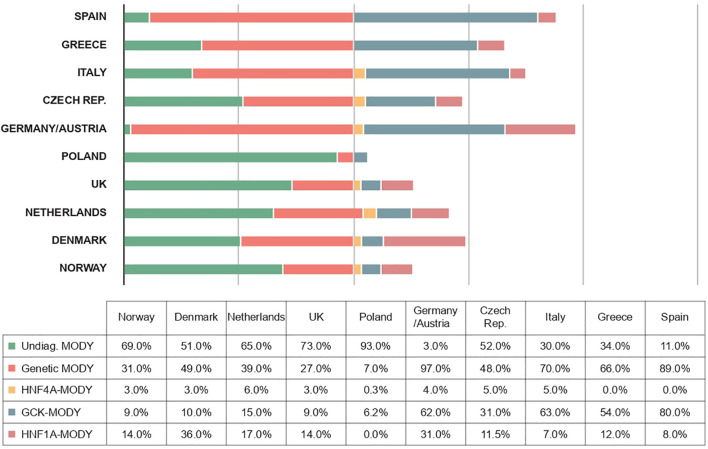
Frequencies of different forms of MODY diabetes in different European populations (1, 19, 100–106).

The frequencies of genetically confirmed MODY, as well as the proportions of different forms of the disease, vary somewhat more between studies depending on the diagnostic criteria used in a given study - for example, when patients are recruited who are negative for autoantibodies (40, 67, 68), the number of cases with an inherited etiology increases dramatically in this cohort (**Figure 3**). Although the prevalence of MODY in regions outside of Europe remains poorly understood, available data from articles suggest geographic and ethnic differences. In the United States, for example, the prevalence of MODY is 1.2% of all pediatric diabetes mellitus cases, and the minimum prevalence of monogenic diabetes in young people under 20 years of age is estimated to be 21 per 1,000,000 (2). On the other hand, in Western Australia, the prevalence of MODY in diabetic patients under 35 years of age is 0.24%, which corresponds to an estimated minimum prevalence of 89 cases per 1,000,000 for the entire Australian population (61).

Unfortunately, the prevalence of MODY in populations in Africa, Asia, South America and the Middle East is unknown. Clearly, studies are needed to elucidate the exact prevalence of MODY in non-European regions. A recent study in Brazil described the first case of NEUROD1-MODY in Latin America and identified a novel frameshift mutation (69), suggesting that studies in countries with multiethnic populations may improve the current understanding of the epidemiology and pathogenesis of MODY. In comparison to European populations, data on the diagnosis of monogenic diabetes in Asian countries are rather scarce (69). A recent study of 82 patients with type 1 diabetes and negative pancreatic cell autoantibodies showed that MODY-HNF1A was the most common form in Chinese patients, but several rare recessive Wolfram syndrome 1 (*WFS1*) mutations were also identified (70). Among Japanese patients with MODY, mutations in *GCK*, *HNF1A*, *HNF4A*, and *HNF1B* genes were detected in 39.2%, of which the proportion of *GCK* gene mutations was 21.6% (71). In Korean patients, the most common form of MODY was *GCK* gene lesions (50%), followed by *HNF1A* (21.4%) and *HNF4A* (21.4%) genes (72).

## Approaches to gene therapy for inherited carbohydrate-metabolizing disorders

The existing literature indicates the significance of molecular genetic testing, which can assist in the selection of the most suitable treatment to optimize blood glucose control, reduce the risk of hypoglycemic events and long-term complications, and ensure appropriate genetic counseling of patients. The physician determines the most appropriate treatment tactics, which may include medication, insulin administration, or dietary correction, based on the specific genetic variant. The most prevalent forms of MODY in individuals with mutations in the *HNF4A* and *HNF1A* genes are specified by specific beta-cell dysfunction. Patients with *HNF1A*-diabetes exhibit reduced insulin secretory capacity, while those with *GCK*-diabetes display defective glucose sensitivity but retain insulin secretory capacity. Patients with MODY are effectively treated with sulfonylureas due to their high sensitivity to these drugs. However, they are also prone to developing hypoglycemia over time. Patients with mutations in the *GCK* gene typically do not require treatment, while oral hypoglycemic drugs are recommended for patients with a confirmed diagnosis and mutations in the *HNF4A* and *HNF1A* genes. A review of recent data indicates that agents that lower blood sugar, in addition to traditional sulfonylurea drugs, may be effective in patients with MODY (73). A number of recommendations have been formulated for the appropriate management of pregnancy in individuals carrying the *GCK* mutation, given that the disruption of carbohydrate metabolism during this period can have a detrimental impact on fetal health. In all other situations, the optimal approach to correcting carbohydrate metabolism in patients with *GCK* gene damage remains a matter of debate (74). The prevalence of other MODY subtypes is considerably lower, and the limited data on treatment options and variations in clinical guidelines across different countries make it challenging to establish a universal approach to managing these patients. **Supplementary Table S1** presents an overview of the various clinical manifestations of MODY, with a focus on the specific genetic variant associated with each form of the disease (**Supplementary Table S1**).

The efficacy of sulfonylurea therapy for the treatment of these forms of diabetes is well documented. Long-term use of sulfonylurea compounds has been observed to induce an increase in glucose-induced insulin secretion for up to 33 years in some patients with MODY. However, in most cases, glucose-induced insulin secretion has been found to decrease over time. As indicated in the literature, a decline of approximately 1-4% per year has been observed (75). Some patients become insensitive to sulfonylurea after 3-25 years, resulting in a lack of or minimal increase in glucose-induced insulin secretion. This subsequently leads to the requirement for insulin to normalize fasting hyperglycemia. In some patients with MODY, insulin and C-peptide levels may be so low that they resemble those observed in patients with type 1 diabetes, with the same typical labile glucose levels (75, 76). There is a paucity of prospective, randomized, controlled data to support the use of adjuvant or alternative therapies (77). Given the heterogeneous nature of different MODY-diabetes variants and the high prevalence of individual forms of diabetes, it can be postulated that the development of gene therapy products for different forms of diabetes may yield therapeutic success. However, given the high clinical and genetic heterogeneity of hereditary forms of MODY diabetes, the creation of a gene product will be a very challenging and, in some cases, impossible task.

However, in recent years, a relatively large number of gene therapy products have been appearing on the market, driven in part by the identification and introduction of fundamentally new gene delivery vectors. Adeno-associated viruses (AAVs), non-enveloped DNA-containing viruses belonging to the Parvoviridae family that, can be used after modifications as vectors to deliver genetic material to target cells, have attracted considerable attention in this field, especially in experimental therapeutic strategies at the clinical stage (78). AAV vector systems offer the same key advantages as AAV, which makes them a promising platform for gene therapy. AAV vectors are non-pathogenic, exhibit low immunogenicity, and present a low risk of insertional mutagenesis. Following the engineering modifications, during which the rep and cap genes necessary for virus replication and its further assembly are removed and, respectively, replaced by a transgene with a promoter, AAV vectors remain in a non-hazardous episomal form within the nucleus of the host cell (79). Besides, AAVs are capable of maintaining stable and prolonged expression of therapeutic genes in quiescent cells, such as neurons and myocytes, which is crucial for the sustained efficacy of numerous gene therapy strategies. Furthermore, AAV capsid proteins, which encapsulate the AAV genome and determine its tropism and entry into target cells, are highly versatile and amenable to modification. This feature enables the engineering of AAV vectors to possess diverse tropisms, thus facilitating the treatment of a wide range of diseases. The low immunogenicity of AAV vectors in comparison to other vectors is attributed to their inefficient transduction of antigen-presenting cells (APCs) (80), which contributes to the AAV vector’s status as one of the leading platforms for gene therapy delivery (81, 82). Genotherapy, which employs AAV constructs, is employed in the treatment of a diverse array of hereditary pathologies. Apart from the European Medicines Agency (EMA)-approved alipogenin tipervovec (Glybera), the most successful use of an adeno-associated viral construct is a dosage form for gene therapy of hereditary forms of tapetoretinal retinal abiotrophy due to mutations in the RPE65 gene (voretigene neparvovec-RPE65 by Spark Therapeutics) (83). This method of local delivery has been demonstrated to be safe and effective. The sole disadvantage associated with this drug is the potential for difficulty in surgical techniques for vector delivery. Applied Genetic Technologies Corporation (AGTC) is pursuing analogous strategies for the treatment of X-linked retinoschisis and achromatopsia, X-linked retinitis pigmentosa, and age-related macular degeneration. These programs are at different stages of development, with the most advanced ones for X-linked retinoschisis and achromatopsia currently undergoing Phase I safety studies (80, 83–85).

The existing literature on the creation of drugs for gene therapy of carbohydrate metabolism disorders is notably sparse. In general, a significant proportion of publications are devoted to the development of genetic constructs and their testing in mice. Genotherapeutic approaches that modulate the activity of specific genes, including *FGF21*, *BMP7*, *GHR*, *IGF1*, *HNF1A*, *INS*, and *GCK*, have been identified. Consequently, native FGF21 enhances glucose uptake in adipocytes, which is of significant consequence for the interaction of insulin with Glut1 receptors in adipose tissue. When expressed at an optimal level, FGF21 protects animals from obesity induced by a diet rich in fat and carbohydrates. The elevation of FGF21 protein in mice is accompanied by an increase in energy expenditure and lipolysis (86). Additionally, FGF21 exhibits relatively low pharmacokinetic properties, rendering gene therapy an appealing strategy for achieving sustained levels of this protein. In this instance, AAV vectors were employed to genetically engineer the delivery of a genetic construct into the liver, adipose tissue, or skeletal muscle in order to facilitate the secretion of FGF21. The administration of recombinant FGF21 protein to mice fed an ob/ob, db/db, or high-fat diet (HFD), or obese Zucker diabetic fatty (ZDF) rats, has been shown to result in a significant reduction in obesity, lower blood glucose and triglyceride levels, and improved insulin sensitivity (87, 88). The therapeutic effect was achieved without the development of adverse effects, despite persistently elevated serum FGF21 levels. This study underscores the promise of FGF21-based gene therapy for the treatment of obesity, insulin resistance, and various types of diabetes mellitus (DM2).

Bone morphogenic protein 7 (BMP7) has demonstrated an ability to enhance energy expenditure through the induction of thermogenesis, as evidenced in short-term investigations employing recombinant protein or adenoviral vectors encoding *BMP7* in mice. To achieve the long-term effects of *BMP7*, the use of AAV vectors provides sustained production of the protein after a single injection. In this study, the administration of AAV-BMP7 vectors to obese mice resulted in a long-lasting increase in serum levels of the factor in hepatocytes and in white adipose tissue. The results indicated that the concentration of the BMP7 protein was correlated with the degree of darkening of the white adipose tissue, as well as the activation of the brown adipose tissue. The observed increase in energy expenditure, along with a reduction in the size and accumulation of fat in the white adipose tissue, and steatosis, resulted in a normalization of body weight and insulin resistance. The results of this study suggest the potential efficacy of AAV-BMP7-mediated gene therapy for the treatment of insulin resistance, type 2 diabetes, and obesity (89).

A recent study has identified a hepatocyte-specific role in growth hormone receptor (GHR) signalling in the regulation of steatosis. A mouse model with hepatocyte-specific GHR knockdown (aHepGHRkd) was employed, wherein the knockdown was established in adult individuals. To prevent the decline in insulin-like growth factor 1 (IGF1) levels and the subsequent rise in insulin resistance observed in aHepGHRkd mice, subsets of aHepGHRkd mice were treated with AAV vectors that induced hepatocyte-specific expression of IGF1 and a constitutively active form of STAT5B. The effects of hepatocyte-specific modulation of *GHR*, *IGF1*, and *STAT5b* on carbohydrate and lipid metabolism have been studied in different nutritional states and in the context of hyperinsulinemic/euglycemic clamps. These preliminary findings suggest a tentative understanding of the physiological role of growth hormone in metabolic regulation in adults, with the potential to protect against the progression of nonalcoholic fatty liver disease (90).

A mouse model of MODY3 was generated using CRISPR/Cas9 genomic editing technology, which demonstrated the suppression of *HNF1A* protein production in the pancreas. These gene-modified mice displayed β-cell dysfunction and markedly elevated blood sugar levels, which are also observed in patients with genetic abnormalities in *HNF1A*. The animals were injected with AAV vector constructs encoding the *Hnf1α* gene under the control of a β-cell-specific promoter, resulting in the expression of *HNF1A* in islet cells. This, in turn, increased the expression of the core proteins GLUT2 and L-PK. AAV-Hnf1α-mediated gene therapy prevented the development of hyperglycemia, hyperinsulinemia, increased glucose tolerance, and enhanced insulin secretion by the pancreatic islet apparatus, thereby reversing MODY3 in rodents. These results demonstrate the efficacy of gene therapy for a monogenic form of sarcomeric diabetes and provide a rationale for applying AAV-Hnf1α-based gene therapy to treat MODY 3 patients in the future (91).

A gene therapy approach for glycemic control based on the co-expression of insulin and glucokinase genes in canine skeletal muscle was developed. A long-term study in diabetic animals using AAV vectors was conducted. The objective of achieving successful long-term glycemic control was met, with no requirement for the administration of exogenous insulin. This study demonstrated the long-term efficacy and safety of AAV-mediated insulin and glucokinase gene transfer in large animals. Furthermore, the capacity of the animal body to adapt to changes in metabolic needs as the animals matured was demonstrated (92, 93). This is a highly impressive and encouraging result, as this approach can be applied in humans with both different forms of MODY and other monogenic forms of carbohydrate metabolism disorders. As is the case with many clinical trials, it is challenging to ascertain the generalizability of these outcome data to the MODY patient population, particularly in light of the low rates of diagnosis in numerous regions worldwide and the relatively straightforward nature of the specific treatment for this disease (**Supplementary Table S1**). Nevertheless, the rapid advancement of research on the impact of AAV vectors on humans, coupled with the availability of diverse gene therapy strategies for various forms of carbohydrate metabolism disorders in animal models, offers optimism for the development of comprehensive gene therapy agents that could benefit patients with severe carbohydrate metabolism disorders.

In summary, the risk-to-benefit ratio for AVV constructs appears favorable. All parties involved, including physicians and patients, anticipate the imminent availability of a number of new genotherapeutic agents for the treatment of MODY in the pharmaceutical market.

## Conclusion

Maturity-onset diabetes (MODY) is a group of monogenic diseases that result in primary defects in insulin secretion and dominantly inherited forms of non-autoimmune diabetes. Although numerous genes can be associated with monogenic diabetes, it is heterozygous mutations in six of them that are responsible for the majority of MODY cases. Glucokinase (GCK)-MODY is caused by mutations in the glucokinase gene. Three MODY subtypes are associated with mutations in hepatocyte nuclear factor (HNF) transcription factors, and two others are associated with mutations in ABCC8 and KCNJ11, which encode subunits of the ATP-dependent potassium channel in pancreatic beta cells. GCK-MODY and HNF1A-MODY are the most prevalent subtypes. The clinical presentation of MODY subtypes is dependent on the gene involved, and the diagnosis of MODY may be considered in a variety of clinical circumstances. However, with the exception of GCK-MODY patients, whose phenotype is very homogeneous, in most cases the penetrance and expression of this molecular abnormality varies greatly between patients. Furthermore, alterations in different genes may result in similar phenotypes. In addition, it can be difficult to distinguish between the most common forms of diabetes, especially type 2 diabetes. Therefore, MODY is distinguished by a high degree of clinical and genetic heterogeneity.

Given the high genetic and clinical polymorphism of the disease, it is challenging to develop a universal genetic construct that is suitable for different genetic variants of MODY and associated complications of diabetes.

The strategy of gene therapy for monogenic forms of MODY is still an experimental direction. It is unlikely to be widely used in the clinic due to several factors. Firstly, the genetic structure of the disease is complex, with high genetic polymorphism and clinical variability even within the same hereditary form. Secondly, there is a lack of clear gene-phenotypic correlations. Most mutations that are the main cause of MODY have low penetrance, while the effectiveness of therapies such as sulfonylurea derivatives and insulin has resulted in the majority of patients being cured.

To date, a wide range of research is being conducted worldwide to study in detail the pathogenesis of type I and II DM, approaches to differential diagnosis, and the development of approaches to molecular correction of carbohydrate metabolism disorders. The use of different classes of oral hypoglycemic agents in maturity-onset diabetes of the young (MODY) is being widely studied. However, the “gold standard” for the treatment of many forms of this disease remains drugs from the sulfonylurea group and insulin administration in severe hyperglycemic states.

Consequently, a meticulous examination of the family history and clinical presentation of patients suspected of having MODY is essential for the identification of those who require genetic testing. A comprehensive examination of MODY epidemiology is essential for the provision of optimal and expedient medical and genetic counseling. This, in turn, will facilitate the more precise administration of genotherapeutic drugs, taking into account the ethnic background of patients, as the spectrum and prevalence of MODY mutations across different populations are crucial factors in gene therapy. The diagnosis of monogenic diabetes has significant implications for prognosis, therapy, and family screening. Consequently, the approaches to the diagnosis and treatment of this disease remain undefined, which also impedes the development of gene therapy. Moreover, in recent years, the advent of rapidly developing molecular genetic tools has led to the emergence of novel monogenic forms of diabetes mellitus and carbohydrate metabolism disorders, further complicating the accurate and timely diagnosis of this disease.
