# Supplementary material for: Current views on etiology, diagnosis, epidemiology and gene therapy of maturity onset diabetes in the young
Source: Front Endocrinol (Lausanne). 2025 Jan 20;15:1497298. doi: 10.3389/fendo.2024.1497298 (PMC11788143; doi:10.3389/fendo.2024.1497298)
Supplement: Supplementary file 1 [file Table1.docx]

Supplementary Material

Table S1. Diversity of clinical forms of MODY diabetes, features of clinical manifestation and pathogenesis, and genetic polymorphism

| **Subtype/**  **frequency** | **MODY gene/ location/**  **gene function** | **Function in pancreas** | **Features of pathogenesis** | **Clinical features** | **Treatment** | **The most important mutations in the European and Asian populations** | **OMIM** |
| --- | --- | --- | --- | --- | --- | --- | --- |
| **Pathophysiology: β-cell dysfunction** | | | | | | | |
| HNF4A-MODY  or MODY1  5-10% | *HNF4A (20q13.12)*  Transcription factor | Foregut differentiation;  Insulin synthesis and  secretion in mature B cells | HNF4A has been demonstrated to regulate the expression of a number of genes, including hepatocyte nuclear factor 1-alpha (HNF1A) and INS. | Macrosomia, progressive insulin secretory defect, significant increase in fasting glucose levels (above 7.77 mmol/l), progressive deterioration of carbohydrate metabolism, age of onset is 7-9 years, transient neonatal hyperinsulinemic hypoglycemia. | Patients are sensitive to sulfonylurea therapy | c.763C>T (p.Gln255Ter); c.421C>T (p.Arg141Ter); c.340C>T (p.Arg114Trp); NM_175914.4:c.185delc.1052T>G (p.Met351Arg) | [125850](https://omim.org/entry/125850) |
| HNF1B- -MODY  or MODY5  <5% | *HNF1B (17q12)*  Transcription factor | The role of pancreatic development in primitive gut stage and the function of mature beta cells in mature organisms remains unclear. | It regulates the activity of several genes, including HNF4A and PDX1, and plays a role in the regulation of blood glucose levels. | The occurrence of pancreatic hypoplasia and diabetic ketoacidosis in individuals below the age of 10 is rare.  Additionally, the following anomalies were observed: renal anomalies and urogenital tract anomalies. | Insulin | c.529C>T (p.Arg177Ter); c.410_484del (p.Arg137_Lys161del); c.982_986del (p.Pro328fs); c.301G>T (p.Glu101Ter); NM_000458.4:c.477del; c.826C>T (p.Arg276Ter); c.1055dup (p.Tyr352Ter); c.1055dup (p.Tyr352Ter); NM_000458.4:c.544+1G>A; c.443C>G (p.Ser148Trp); c.494G>A (p.Arg165His). | [137920](https://omim.org/entry/137920) |
| NEUROD-MODY  or MODY6  <1% | *NEUROD1 (2q31.3)*  Transcription factor | The typical progression of endocrine cell development and the synthesis and secretion of insulin in fully mature beta cells. | NEUROD1 is responsible for regulating the expression of ABCC8 and GCK genes, as well as the expression of the insulin gene itself. This is achieved by binding to a critical E-box motif on the insulin gene promoter.  Mutations in the NEUROD1 gene that disrupt the DNA-binding domain result in a reduction in E-box binding activity of NEUROD1 or in the production of a truncated polypeptide lacking the C-terminal transactivation domain. This region has been demonstrated to bind to the CBP and p300 coactivators. The genetic defect of NEUROD1 results in impaired development of the C-cells and impaired expression of the glucose transporters (GLUT2) and/or glucokinase. | Ketoacidosis-prone diabetes with microvascular sequelae and neurological abnormalities is a condition that may be overweight or obese, and it is frequently associated with colorectal cancer and polyposis. It presents similarly to severe forms of diabetes mellitus. | Oral antidiabetic agents (OAD) or insulin therapy | c.34G>C (p.Gly12Arg),  c.175G>C (p.Glu59Gln),  c.308G>C (p.Arg103Pro)  c.616dup (p.His206fs) | [606394](https://omim.org/entry/606394) |
| PDX1-MODY  or  MODY4  <1% | *PDX1 (13q12.2)*  Transcription factor | The master regulator of pancreatic formation, differentiation, and maturation of islet cells is responsible for insulin synthesis and secretion in mature β-cells. | PDX1 has been demonstrated to activate HNF1a, HNF1b, and RFX6 during the early developmental stage. However, the regulatory role of KCNJ11 has only been observed subsequent to the formation of the islet.  The phenotypes associated with 33 MODY4-related mutations can be attributed to dysregulation of glucose-stimulated INS promoter activity at the transcriptional level by PDX-1. Additionally, PDX1-binding sites for target genes undergo significant evolution during embryonic development.  The genetic defect of PDX1 results in impaired development of the C-cells and impaired expression of the glucose transporters (GLUT2) and/or glucokinase.  Homozygous and compound heterozygous missense and frameshift mutations are the underlying cause of pancreatic agenesis. | The condition presents with a number of characteristics, including pancreatic agenesis, overweight or obesity in some cases, ketoacidosis-prone diabetes with microvascular sequelae and neurological abnormalities, and severe manifestations of the malabsorption syndrome. It typically manifests at a later age, from 17 to 60 years and older. | The treatment plan may include dietary modifications, oral antidiabetic drugs (OAD), or insulin. | c.670G>A (p.Glu224Lys); c.590G>A (p.Arg197His); c.533A>G (p.Glu178Gly); c.532G>A (p.Glu178Lys); c.492G>T (p.Glu164Asp); | [606392](https://omim.org/entry/606392) |
| PAX4- -MODY  or MODY9    <1% | *PAX4 (7q32.1)*  Transcription  factor | A crucial regulator of pancreatic β-cell development. | It plays a significant role in the differentiation and development of pancreatic islet beta cells. A transcriptional repressor that binds to a common element in the glucagon, insulin, and somatostatin promoters. It engages in competition with PAX6 for the same promoter binding site. | A potential diagnosis is ketoacidosis, hypoplasia, or aplasia of the pancreatic islet apparatus. Additionally, the disease manifests early in life and is characterized by the absence of distinctive clinical features. | The treatment plan may include dietary modifications, oral antidiabetic drugs (OAD), or insulin. | c.514C>T (p.Arg172Trp)  NM_001366110.1(PAX4):c.772-1G>A  c.109C>T (p.Arg37Trp)  c.361C>T (p.Arg121Trp)  c.490C>T (p.Arg164Trp) | [612225](https://omim.org/entry/612225) |
| **Pathophysiology: β-cell dysfunction (decreased glucose sensitivity of β-cell)** | | | | | | | |
| KLF11- -MODY  or  MODY7  <1% | *KLF11 (2p25.1)*  Kruppel-like transcription factors | The typical progression of endocrine cell development | Some mutations have been observed to reduce the binding activity of the KLF11 protein, which has been demonstrated to impair the activation of the insulin gene promoter and lead to a reduction in insulin expression levels by islet cells. This may contribute to the development of diabetes. | The disease is similar to type 2 diabetes mellitus in that it is characterized by an early onset and the absence of distinctive clinical features. | The treatment plan may include dietary modifications, oral antidiabetic drugs (OAD), or insulin. | c.1039G>T (p.Ala347Ser)  c.659C>T (p.Thr220Met) | [610508](https://omim.org/entry/610508) |
| **Pathophysiology: β-cell dysfunction (glucose sensing defect)** | | | | | | | |
| GCK- -MODY  or MODY2  30%-50% | *GCK (7p13)*  Glucose-sensing and  phosphorylating enzyme | Glucokinase (GCK) is responsible for initiating the utilization of glucose and functions as a glucose sensor by regulating the phosphorylation of glucose in beta cells. | In the pancreas, this enzyme is involved in the process of glucose-stimulated insulin secretion.  A reduction in enzyme activity is a consequence of mutations in the GCK gene.  The conversion of glucose into glucose-6-phosphate is absent or reduced, which in turn reduces the production of ATP in mitochondria. This results in the closure of ATP-sensitive potassium channels and the opening of voltage-gated calcium channels. In the absence of calcium, insulin is not released from the β cell. | Stable mild fasting hyperglycemia at birth is typically asymptomatic and may persist for years. Prolonged low fasting glucose (5-8.5 mmol/L) is a hallmark of MODY-2, with the minimum age of onset being the first year of life. The disease may manifest at any age up to 25 years. | In most cases, no medications are typically administered, except during pregnancy. | c.835G>T (p.Glu279Ter); c.683C>T (p.Thr228Met); c.781G>A (p.Gly261Arg); c.895G>C (p.Gly299Arg); NM_000162.5:c.483+2_483+16del; c.391T>C (p.Ser131Pro); c.793G>T (p.Glu265Ter); c.629T>A (p.Met210Lys); c.1132G>A (p.Ala378Thr) | [125851](https://omim.org/entry/125851) |
| **Pathophysiology: β-cell dysfunction (Insulin gene mutation)** | | | | | | | |
| INS- -MODY  or  MODY10  <1% | *INS (11p15.5)*  Encode the proinsulin precursor | A hormone that plays a regulatory role in glucose metabolism within the organism. | Cysteine substitutions that disrupt disulfide bond formation (B7-A7, B19-A20, and A6-A11) result in the retention of both mutant and wild-type proteins in the endoplasmic reticulum (ER). This is due to an improper interaction between the unpaired cysteine of the mutant protein and the wild-type molecule, which subsequently impairs β-cell proliferation through the induction of endoplasmic reticulum (ER) stress. Furthermore, non-cysteine substitutions have the potential to disrupt the normal folding of proinsulin. Mutations in the signal peptide of preproinsulin result in the failure of the peptide to be recognized by signal peptidases, thereby preventing its subsequent translocation into the endoplasmic reticulum. Moreover, certain mutations may impede the binding of insulin to its receptor. Heterozygous mutations of the INS gene have been observed to impair the folding of the proinsulin molecule and to induce apoptosis of β-cells within the endoplasmic reticulum. Mutations that damage critical regions of the preproinsulin molecule have the effect of disrupting the quaternary structure of proinsulin, as well as its posttranslational modifications and insulin secretion. | The condition is characterized by the absence of distinctive clinical features and an early onset.  Additionally, the patient presented with permanent neonatal diabetes mellitus (PNDM). | The treatment plan may include dietary modifications, oral antidiabetic drugs (OAD), or insulin. | c.265C>T (p.Arg89Cys)  c.266G>T (p.Arg89Leu)  c.16C>T (p.Arg6Cys)  c.137G>A (p.Arg46Gln) | [613370](https://omim.org/entry/613370) |
| HNF1A- -MODY  or  MODY3  30%-65% | *HNF1A (12q24)*  Transcription factor | The process of foregut differentiation is essential for the synthesis and secretion of insulin in mature beta cells. | HNF1a binds to the promoters of various genes, including PDX1, INS, and GLUT-2, and regulates glucose sensing, mitochondrial metabolism, insulin secretion, and exocytosis in β-cells. Heterozygous mutations result in impaired insulin synthesis and secretion, progressive β-cell dysfunction, and the presence of a defect in sodium-glucose cotransporter type 2 (SGLT2) due to the expression of HNF1A in renal tubular cells. | Transient neonatal hyperinsulinemic hypoglycemia, progressive insulin secretory defect, renal glycosuria, and higher HDL-cholesterol and lower LDL-cholesterol levels are often accompanied by transient neonatal hypoglycemia and macrosomia. Late onset, rapidly progressive development of the disease and marked glycemia exhibit a more severe course and often develop complications of diabetes, especially retinopathy. | Patients are sensitive to sulfonylurea therapy | c.872dup (p.Gly292fs); c.1340C>T (p.Pro447Leu); c.876del (p.Pro293fs); c.365A>G (p.Tyr122Cys); NG_011731.2:g.4741A>C; c.815G>A (p.Arg272His); c.1747C>G (p.Arg583Gly); c.1859C>T (p.Thr620Ile); NM_000545.8:c.-119del; c.1720= (p.Ser574=); c.1748G>A (p.Arg583Gln); c.335C>T (p.Pro112Leu); c.391C>T (p.Arg131Trp); NM_000545.8:c.587_590del; NM_000545.8:c.714-1G>A; c.827C>A (p.Ala276Asp); NM_000545.8:c.1333_1334del; c.1592G>C (p.Ser531Thr); c.92G>A (p.Gly31Asp) | [600496](https://omim.org/entry/600496) |
| BLK- -MODY  or MODY11  <1% | *BLK (8p23.1)*  Tyrosine kinase functions in signal transduction | The development and secretion of normal endocrine cells in mature β-cells | BLK functions as a stimulator of insulin synthesis and secretion in pancreatic β-cells through the transcription factors Pdx1 and Nkx6.1 in response to glucose.  The overexpression of BLK has been demonstrated to markedly enhance insulin secretion at elevated glucose concentrations. | The patient presents with a diagnosis of overweight/obesity and a relative insulin secretion defect. There is an early onset of the condition, and the absence of distinctive clinical features. | The treatment plan may include dietary modifications, oral antidiabetic drugs (OAD), or insulin. | NC_000008.11:g.11573132C>T NC_000008.11:g.11474238G>A  NM_001715.2(BLK):c.*505G>T  c.211G>A (p.Ala71Thr) | [613375](https://omim.org/entry/613375) |
| APPL1- -MODY  or  MODY14  <1% | *APPL1 (3p14.3)*  Adaptor protein | The process of insulin synthesis and secretion in mature β-cells | The APPL1 gene encodes a protein that directly interacts with adiponectin receptors, thereby mediating adiponectin signaling and its effect on the insulin-signaling pathway. Furthermore, APPL1 mediates other signaling pathways by directly interacting with membrane receptors or signaling proteins, thereby playing a critical role in cell proliferation, apoptosis, cell survival, endosomal trafficking, and chromatin remodeling. | Some subjects exhibited overweight/obesity, dysmorphic phenotype, and developmental delay. Additionally, early onset and the absence of distinctive clinical features were observed. | The treatment plan may include dietary modifications, oral antidiabetic drugs (OAD), or insulin. | c.280G>A (p.Asp94Asn) c.1655T>A (p.Leu552Ter) | [616511](https://omim.org/entry/616511) |
| **ATP-sensitive potassium channel dysfunction** | | | | | | | |
| KCNJ11- -MODY  or MODY13   <1% | *KCNJ11 (11p15.1)*  Subunit of ATP-sensitive channels | The process of β-cell excitation is a function of ATP-sensitive channels. | The protein encoded by this gene is a type of integral membrane protein and inward-rectifier potassium channel. The protein encoded by this gene has a greater tendency to allow potassium to flow into a cell rather than out of a cell. Its activity is controlled by G-proteins and it is found associated with the sulfonylurea receptor SUR.  ATP-gated potassium channels play a significant role in the regulation of insulin secretion, which in turn helps to control blood sugar levels. The closing of the channels in response to elevated glucose levels initiates the release of insulin from beta cells, whereas opening inhibits this process.  Structural integrity is a prerequisite for effective channel behavior, which may be disrupted by mutations through compression of the ion-conducting part of the channel or the cessation of intersubunit interactions. Mutations that result in disconnection between Kir6.2 and SUR1 have the effect of decreasing the ATP sensitivity of the channel.  Mutations in this gene are associated with a condition characterized by unregulated insulin secretion. | Neonatal diabetes can be classified as transient or permanent. Additionally, some cases of neonatal diabetes are associated with overweight or obesity. In some cases, homozygotes may also develop neonatal diabetes. | It is recommended that patients undergo therapy with sulfonylurea drugs and insulin. | c.67A>G, p.Lys23Glu  c.679G>A (p.Glu227Lys)  c.124T>C (p.Cys42Arg) | [616329](https://omim.org/entry/616329) |
| ABCC8- -MODY  or MODY12  <1% | *ABCC8 (11p15.1)*  Subunit of ATP-sensitive channels | The process of β-cell excitation is a function of ATP-sensitive channels. | The sulfonylurea receptor is capable of detecting alterations in ATP and ADP concentrations, thereby influencing the activity of the K (ATP) channel and, in turn, regulating insulin release.  Mutations in this gene are associated with the development of unregulated insulin secretion.  The mutations alter the protein structure, impede channel closure, and result in diminished insulin secretion from beta cells and compromised glycemic control.  The activation of hypothalamic K-ATP channels typically serves to inhibit hepatic gluconeogenesis. Consequently, any alteration in this central nervous system/liver circuitry may potentially contribute to the development of diabetic hyperglycemia. | As with MODY1 and MODY3, the homozygote results in permanent neonatal diabetes, while the heterozygote results in transient neonatal diabetes. | The treatment plan may include dietary modifications, oral antidiabetic drugs (OAD), or insulin. | c.4055G>A (p.Arg1352His) c.4135C>T (p.Arg1379Cys) | [600509](https://omim.org/entry/600509) |
| **Pancreatic endocrine and exocrine dysfunction** | | | | | | | |
| CEL- -MODY  or  MODY8  <1% | *CEL (9q34.13)*  Lipolytic enzyme | The pancreatic exocrine enzyme carboxylester lipase is secreted into the digestive tract. | In acinar cells, the mutant cell enzyme results in endoplasmic reticulum (ER) retention and the activation of the unfolded protein response (UPR). The acinar cells are unable to overcome the excess amount of misfolded protein. Modified misfolded CEL protein gains access to the extracellular space through the induction of exocytosis, where it subsequently forms insoluble aggregates. Cross-talk between acinar and islet cells enables the insoluble aggregates to be re-uptaken by neighboring β-cells via endocytosis. Subsequently, the capacity of β-cells to degrade endocytic substrates through lysosomal pathways is depleted. The interaction of CEL aggregates with cell membranes results in the dysfunction of various organelles, as evidenced by alterations in mitochondrial activity and the onset of ER stress. | Exocrine insufficiency, lipomatosis: The pancreas may exhibit a number of pathological changes, including lipomatosis, fibrosis, and atrophy, as well as exocrine insufficiency. | The treatment plan may include dietary modifications, oral antidiabetic drugs (OAD), or insulin. | c.703C>T (p.Arg235Ter)  c.1776dup (p.Val593fs)  c.1776del (p.Val593fs) | [609812](https://omim.org/entry/609812) |
